# Calcitriol reduces kidney development disorders in rats provoked by losartan administration during lactation

**DOI:** 10.1038/s41598-017-11815-8

**Published:** 2017-09-13

**Authors:** Lucas Ferreira de Almeida, Heloísa Della Coletta Francescato, Cleonice Giovanini Alves da Silva, Roberto Silva Costa, Terezila Machado Coimbra

**Affiliations:** 1 Department of Physiology of Ribeirão Preto Medical School, University of São Paulo, Ribeirão Preto, São Paulo Brazil; 2 Department of Pathology of Ribeirão Preto Medical School, University of São Paulo, Ribeirão Preto, São Paulo Brazil

## Abstract

Calcitriol has important effects on cellular differentiation and proliferation, as well as on the regulation of the renin gene. Disturbances in renal development can be observed in rats exposed to angiotensin II (AngII) antagonists during lactation period. The lack of tubular differentiation in losartan-treated rats can affect calcitriol uptake. This study evaluated the effect of calcitriol administration in renal development disturbances in rats provoked by losartan (AngII type 1 receptor antagonist) administration during lactation. Animals exposed to losartan presented higher albuminuria, systolic blood pressure, increased sodium and potassium fractional excretion, and decreased glomerular filtration rate compared to controls. These animals also showed a decreased glomerular area and a higher interstitial relative area from the renal cortex, with increased expression of fibronectin, alpha-SM-actin, vimentin, and p-JNK; and an increased number of macrophages, p-p38, PCNA and decreased cubilin expression. Increased urinary excretion of MCP-1 and TGF-β was also observed. All these alterations were less intense in the losartan + calcitriol group.The animals treated with calcitriol showed an improvement in cellular differentiation, and in renal function and structure. This effect was associated with reduction of cell proliferation and inflammation.

## Introduction

Kidney development is a highly complex process that requires the precise regulation of cellular proliferation, differentiation, and apoptosis^1^. Human kidney development is completed in utero by week 36^2^, whereas in the rat, nephrogenesis continues into the second postnatal week^3^. Rats are born with immature kidneys, and the first 2 postnatal weeks correspond to the second and third trimesters of kidney development in the human fetus. Therefore, the neonatal rat model seems useful for studying the mechanisms of kidney development in the human fetus^4^. Several experimental and clinical studies demonstrated that the renin-angiotensin system (RAS) participates in renal development^5, 6^. Nadeem *et al*.^7^ described the presence of acute kidney injury, chronic kidney disease, and tubular dysfunction in children with a history of intrauterine exposure to RAS blockers. Several studies have demonstrated abnormal renal development in rats following neonatal treatment with antagonists of the RAS. These studies showed an increase in the relative interstitial area from the renal cortex^8, 9^, increased apoptosis, decreased cell proliferation^5^, and impaired expression of growth factors in the kidneys after RAS inhibition^10^. Chen *et al*.^11^ observed that the treatment of neonatal rats with losartan for 2 days provoked downregulation of genes encoding cytoskeletal and extracellular matrix ECM components, resulting in ECM malformation and cell-cell and cell-matrix interaction dysfunctions.

Disturbances in renal development have been associated with an increase in anti-α smooth muscle actin (α-SMA), vimentin, and fibronectin expression in renal tissue^5–12^ and a decrease of the proximal tubule cubulin receptor^13^, which characterizes the epithelial- mesenchymal transition (EMT) process. EMT is a physiological process that occurs during early embryogenesis, tissue repair, and pathology^14^. The epithelial cells only express alfa-SMA and vimentin before of the differentiation or in the transdifferntiation processes. During this process these cells can proliferate, migrate and produce extracellular matrix. Therefore, these proteins can be used as a marker of cell indiferentiation.

Calcitriol is mostly known for its role in the regulation of calcium homeostasis and bone metabolism^15, 16^. However, recent studies have shown that calcitriol is also involved in the homeostasis of several other cellular processes, including the modulation of autoimmunity, inflammatory process, and control of blood pressure^17–19^. It also participates in the regulation of cell proliferation and differentiation processes^15^ and in the regulation of the renin gene^20^.

In the present study, we evaluated the influence of calcitriol treatment on inflammation, cell differentiation and proliferation, and the disturbances of renal function and structure in rats exposed to losartan during lactation.

## Results

### Body weight and water and food consumption

No difference was observed in body weight and in water and food consumption among all dam groups during lactation. The body weight gain of the offspring from the losartan group during the lactation period (from birth to 21 days of age) was lower compared to the control group in the second and third weeks. At this time, we also observed an increase in mortality (40%) in the pups of the losartan group. No mortality was observed in the groups in 21 and 60 days of age. Higher water ingestion was observed in both pup groups exposed to losartan compared to the control groups. Treatment with calcitriol had no effect on this increase. Food consumption was similar among the animals of the different groups: control (40.23 ± 0.89), control + calcitriol (42.76 ± 0.23), losartan (39.32 ± 0.76), and losartan + calcitriol (37.45 ± 0.34). Data are expressed as mean ± SEM. n = 9–14 animals per group.

### Studies of renal function

The results of the renal function parameters of the animals at the end of the experimental period (60 days) are shown in Table 1. The creatinine clearance was reduced in the losartan-treated pups. This animal group also showed increased albuminuria, and higher fractional excretion of sodium (FE_Na+_) and potassium (FE_K+_), compared to the controls. Calcitriol treatment attenuated the increased FE_Na+_, FE_K+_, and albuminuria and prevented the decrease in the creatinine clearance provoked to losartan. Compared to controls, higher urine volume and lower osmolality were observed in the groups of rats exposed to losartan. Calcitriol treatment did not affect these parameters. The losartan group also had higher systolic blood pressure (SBP) compared to the control groups, which was attenuated by calcitriol treatment (Table 1).

**Table 1:** Systolic blood pressure (SBP), albuminuria (ALB), 24-h urine volume (V), urinary osmolality (U), glomerular filtration rate (GFR), and fractional excretions of sodium (FE_Na+_) and potassium (FE_K+_) in 60-day-old pups from the control and losartan-treated groups.

	Groups			
	control	control + calcitriol	losartan	losartan + calcitriol
SBP (mmHg)	120 ± 1.3	117 ± 1.4	140 ± 0.8***^,###^	129 ± 0.7***^,###,§§§^
ALB (mg 24 h^−1^)	0.5 (0.4; 0.6)	0.4 (0.4; 0.6)	7.9 (6.8; 9.6)***^,###^	4.9 (4.2; 5.6)**^,###,§§§^
V (ml)	14.5 ± 0.79	13.88 ± 1.66	35.9 ± 3.21***^,###^	33.6 ± 3.23***^,###^
U (mOsm kg H_2_0^−1^)	1.578 ± 75	1.612 ± 121	703 ± 25***^,###^	791 ± 32***^,###^
GFR (ml min^−1^100 g^−1^)	0.88 ± 0.07	0.89 ± 0.04	0.56 ± 0.12*^,#^	0.77 ± 0.16*^,#,§^
FE_Na+_ (%)	0.15 (0.1; 0.3)	0.14 (0.1; 0.3)	0.55 (0.3; 0.8)**^,##^	0.43 (0.4; 0.9)*^,#,§^
FE_K+_ (%)	27.2 (21.3; 36.1)	24.3 (28.3; 58.7)	88.1 (44.9; 114.4)*^,#^	69.4 (36.8; 105.1)*^,#,§^

Data are expressed as median and interquartile range (25^th^–75^th^) (ALB, FE) or mean ± SEM (SBP, V, U, GFR). n = 7–12 animals per group. *P < 0.05, **P < 0.01 and ***P < 0.001 vs. control; ^#^ *P* < 0.05, ^##^ *P* < 0.01 and ^###^ *P* < 0.001 vs control + calcitriol; and ^§^ *P* < 0.05, and ^§§§^ *P* < 0.001 vs. losartan.

### Histologic and morphometric analysis

**Figure 1:**
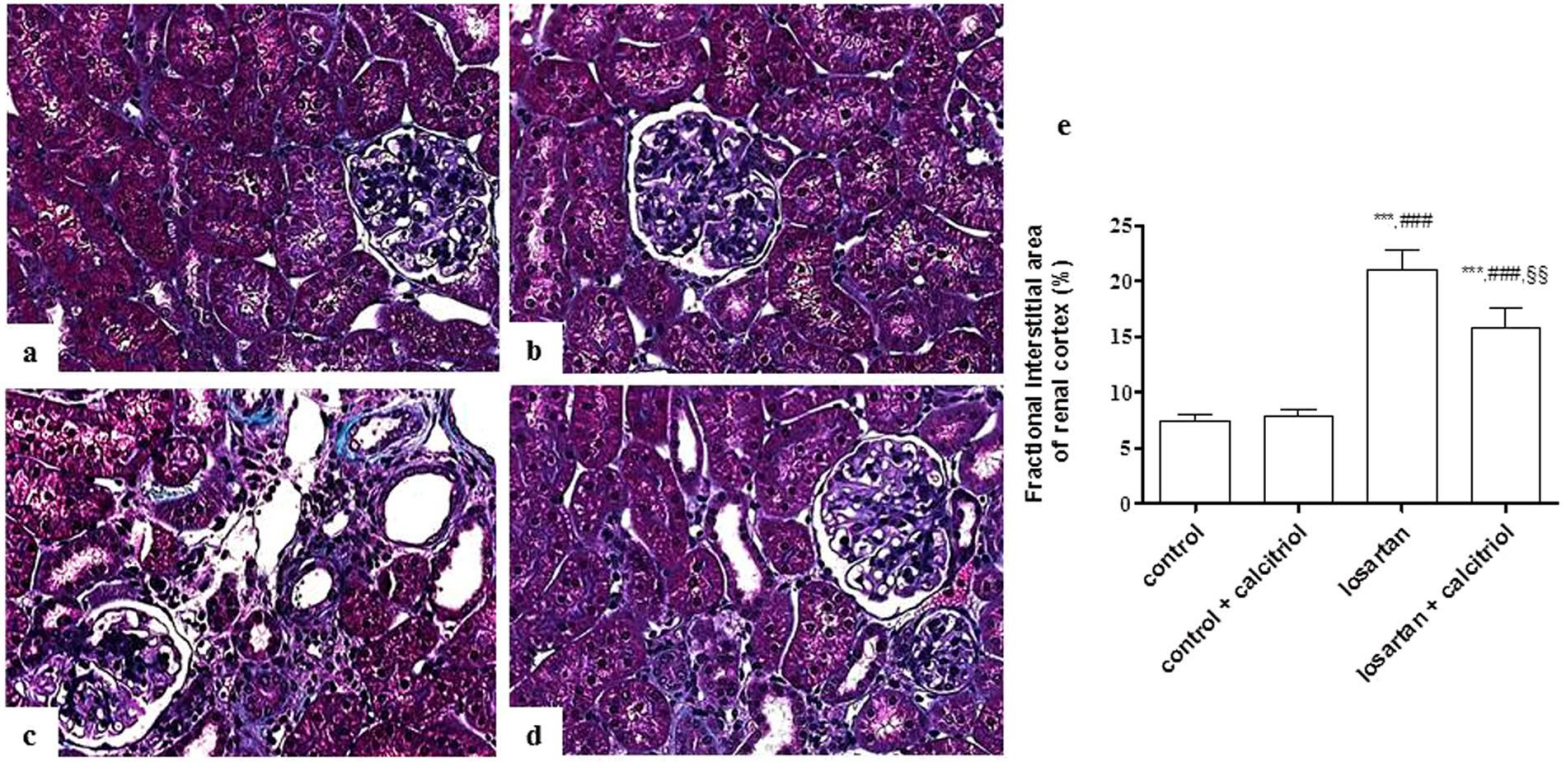
Representative Masson’s trichrome-stained histological sections from the control (**a**), control + calcitriol (**b**), losartan (**c**), and losartan + calcitriol (**d**) groups at 60 days of age. Note that the interstitial lesions in (**c**) are more intense than in (**d**). Scale bar = 50 μm. (**e**) Morphometric analysis of the fractional interstitial area (%) from all groups. The data are expressed as mean ± SEM. n = 7–12 animals per group. ***P < 0.001 vs. control; ^###^ *P* < 0.001 vs. control + calcitriol; and ^§§^ *P* < 0.01 vs. losartan.

**Figure 2:**
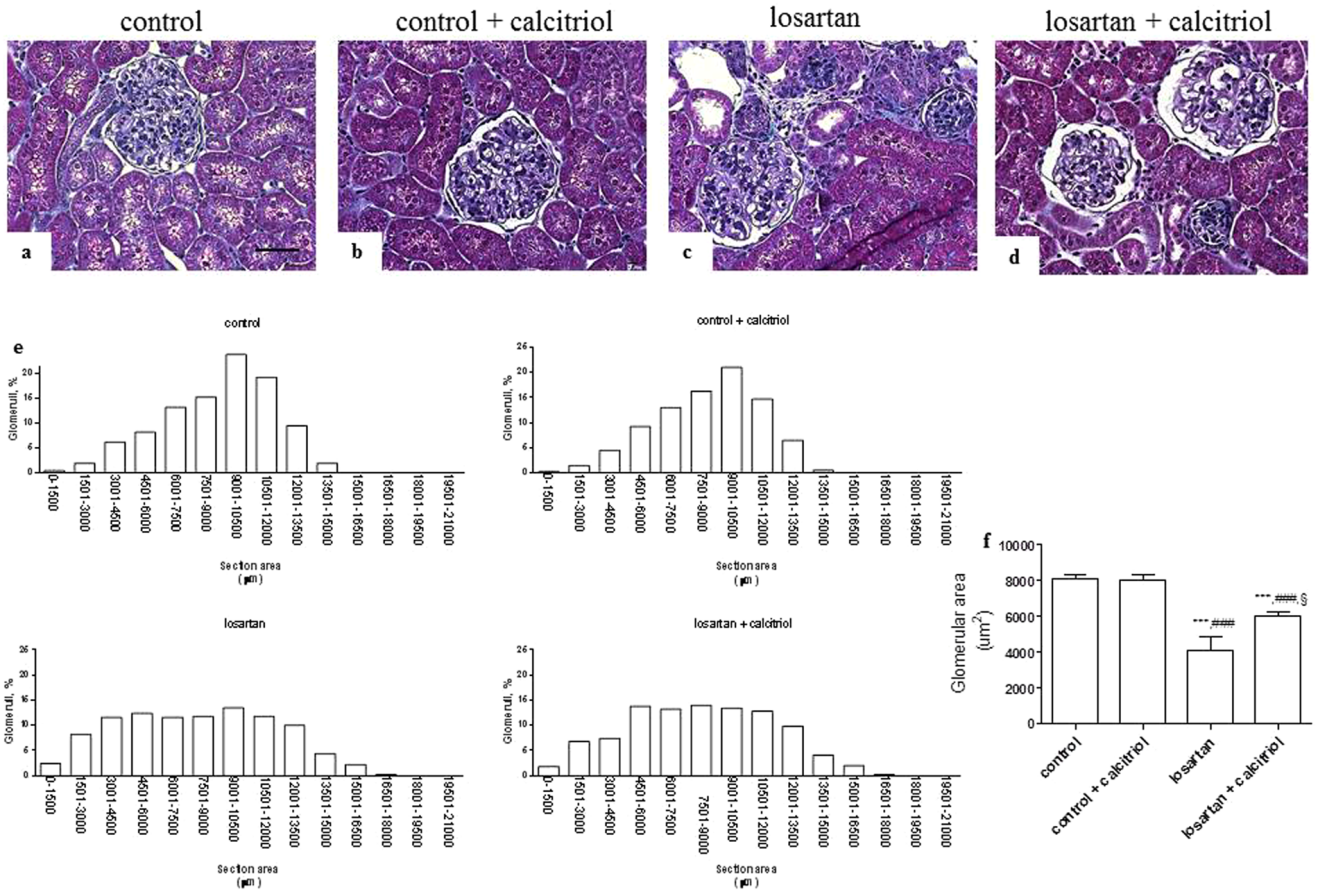
Histological sections stained with Masson’s trichrome showing the distribution of the glomerular area from the control (**a**), control + calcitriol (**b**), losartan (**c**), and losartan + calcitriol (**d**) groups at 60 days of age. Scale bar = 50 μm. The population of smaller glomeruli is evidenced in the losartan group. (**e**) The frequency distribution of the glomerular areas in the groups. Non-Gaussian distribution is observed in the losartan group, and a subpopulation of smaller glomeruli in losartan and losartan + calcitriol can be observed. (**f**) Mean glomerular area of the control and experimental groups. Scale bar = 50 μm. Data are expressed as mean ± SEM. n = 7–12 animals per group. ***P < 0.001 vs. control; ^###^ *P* < 0.001 vs. control + calcitriol; and ^§^ *P* < 0.05 vs. losartan.

The morphometric analysis of the cortical relative interstitial area is shown in Fig. 1. Losartan treated rats presented tubules with dilated lumens, loss of the brush border and tubular epithelial flattening, as well as an increase in the relative interstitial area with infiltration of inflammatory cells, and fibrotic areas (Fig. 1c). These alterations were attenuated by treatment with calcitriol (Fig. 1d). The results of the studies regarding the frequency of distribution of the glomeruli, according to their areas, are shown in Fig. 2. In the control groups (Fig. 2a and b) a frequency plot was observed that shows a normal distribution (Gaussian curve) of the glomerular area (Fig. 2e). This group showed an increase in the percentage of glomeruli with smaller areas (Fig. 2c and d) compared to the control groups, and a non-Gaussian distribution was obtained (Fig. 2e). In the control groups the frequency of glomeruli with a glomerular area of 4,500 μm^2^ was about 8% of the total evaluated glomeruli. A higher frequency of these glomeruli was observed in the animals of the losartan group. The animals in the losartan group also had a higher number of hypertrophied glomeruli (Fig. 2e). Although the animals of the losartan group had a higher number of hypertrophied glomeruli, the mean glomerular area was smaller, due to the smaller proportion of glomeruli being larger. The increase of the number of the hypertrophied glomeruli can be a mechanism compensatory to the decrease of the glomerular area mean for kidney. The mean glomerular area was 7,900 μm^2^ ± 467.18 μm^2^ in the control group; 7,789 μm^2^ ± 321.11 μm^2^ in the calcitriol control group; 4,917 μm^2^ ± 213.31 μm^2^ in the losartan group; and 5,946 μm^2^ ± 231.34 μm^2^ in the calcitriol + losartan group (Fig. 2f).

### Immunohistochemical studies

**Figure 3:**
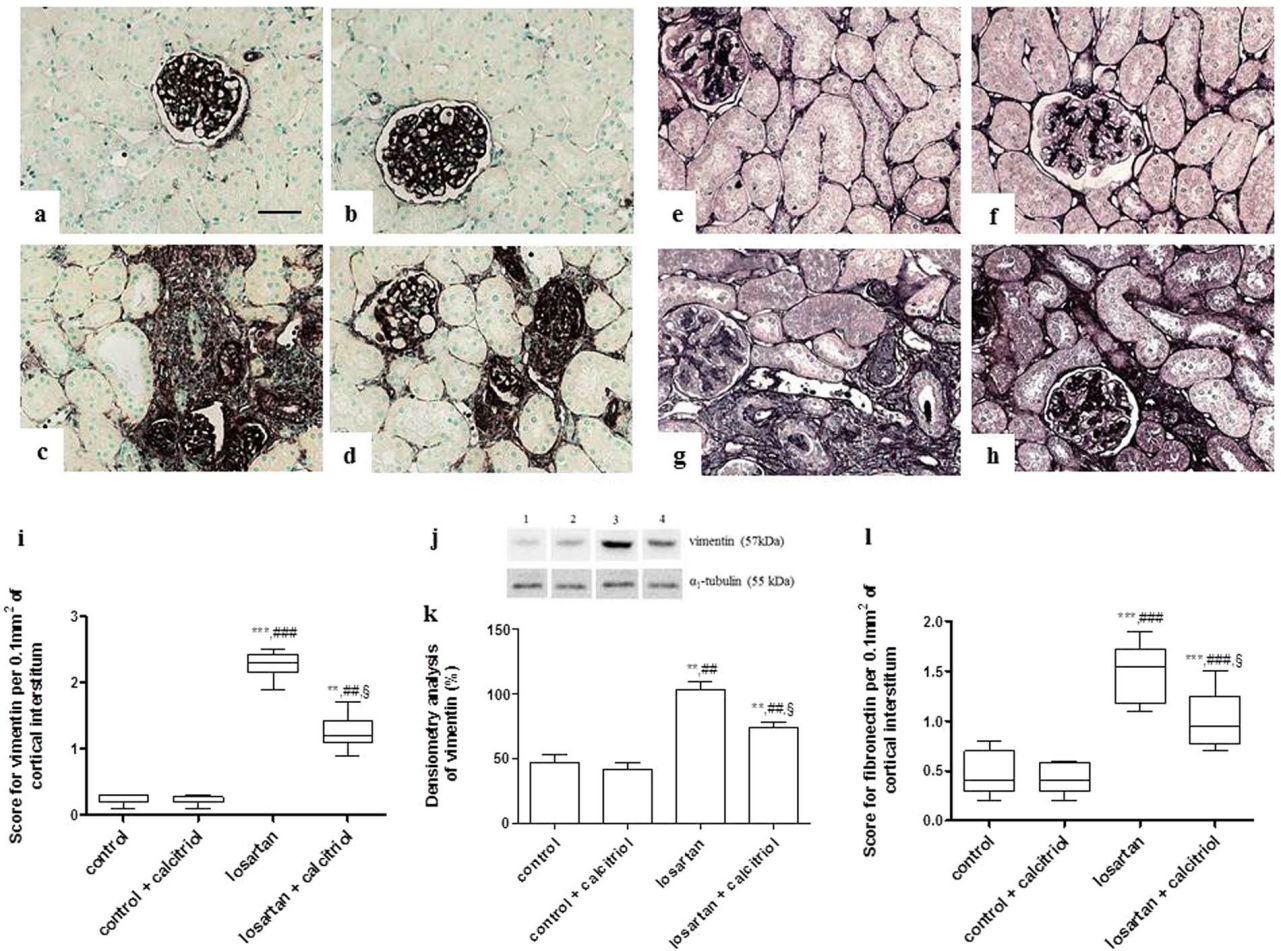
Immunolocalization of vimentin (**a**–**d**) and fibronectin (**e**–**h**) in the renal cortex of the control (**a**,**e**), control + calcitriol (**b**,**f**), losartan (**c**,**g**), and losartan + calcitriol (**d**,**h**) groups. Scores for vimentin (**i**) and fibronectin (**l**) in the renal cortex. Scale bar = 50 μm. Western blot analysis of vimentin and α1- tubulin (**j**) in renal cortex from control (lane 1), control + calcitriol (lane 2), losartan (lane 3) and losartan + calcitriol (lane 4) groups. Densitometry of vimentin (**k**). A densitometric ratio between the densitometry of vimentin and α1-tubulin was calculated and data are expressed in comparison to the control, with the mean (±SEM) control value designated as 100%. Blots are representative images from independent experiments (n = 5–6 for each group). Immunohistochemical data are expressed as median and interquartile range (25^th^–75^th^) (i and j) and Western blot data are expressed as mean ± SEM (k). **P < 0.01 and ***P < 0.001 vs. control; ^##^ *P* < 0.01 and ^###^ *P* < 0.001 vs. control + calcitriol; and ^§^ *P* < 0.05 vs. losartan.

**Figure 4:**
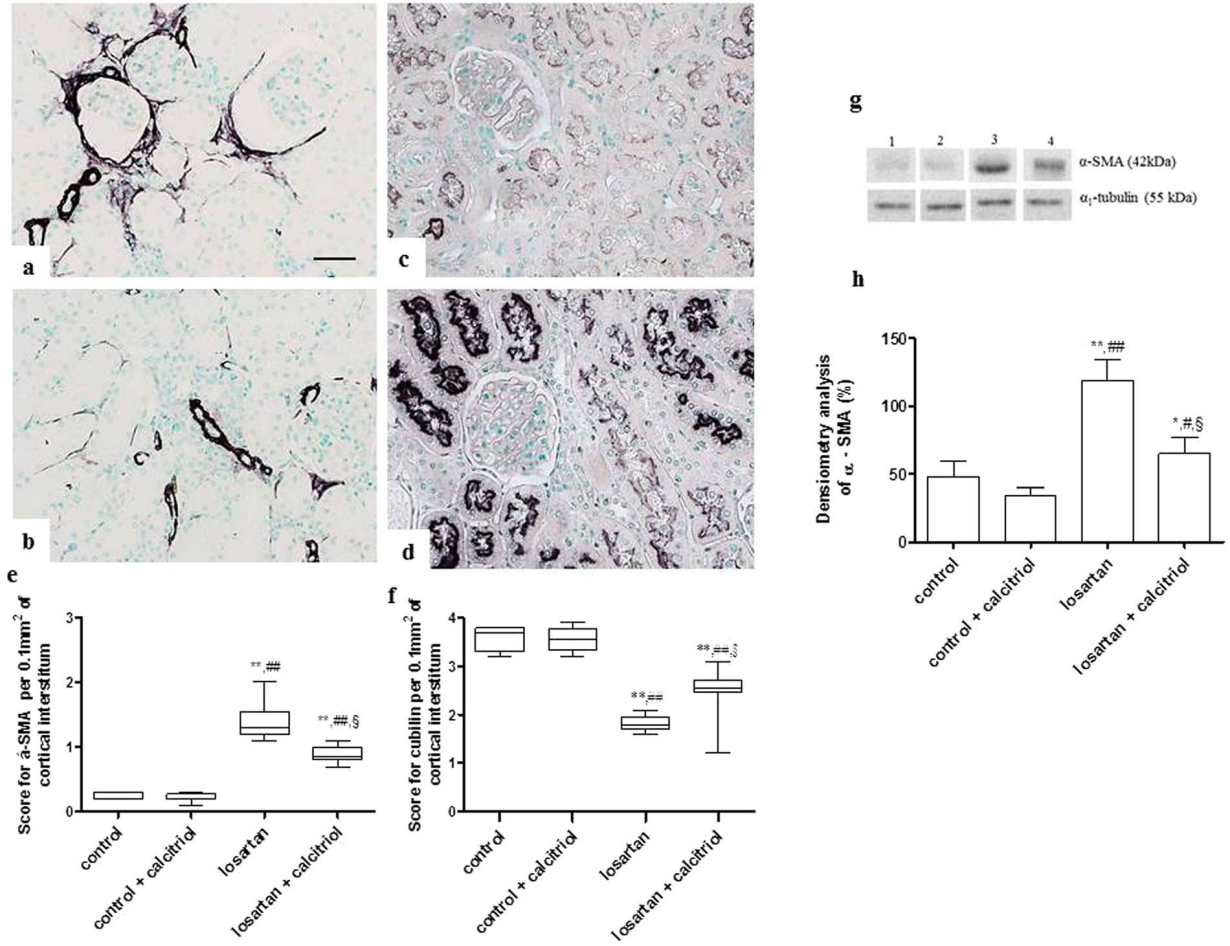
Immunolocalization of α-SMA in the renal cortex from the losartan (**a**), losartan + calcitriol (**b**), and immunolocalization of cubilin from the losartan (**c**), and losartan + calcitriol (**d**) groups. Scores for α-SMA in the tubulointerstitial area (**e**) and cubilin (**f**) in the renal cortex. Scale bar = 50 μm. Western blot analysis of α-SMA and α1- tubulin (**g**) in renal cortex from control (lane 1), control + calcitriol (lane 2), losartan (lane 3) and losartan + calcitriol (lane 4) groups. Densitometry of α-SMA (**h**). A densitometric ratio between the densitometry of α-SMA and α1-tubulin was calculated and data are expressed in comparison to the control, with the mean (±SEM) control value designated as 100%. Blots are representative images from independent experiments (n = 5–6 for each group). Immunohistochemical data are expressed as median and interquartile range (25^th^–75^th^) and Western blot data are expressed as mean ± SEM (**h**). *P < 0.05, **P < 0.01, and ***P < 0.001 vs. control; ^#^ *P* < 0.05 and ^##^ *P* < 0.01 vs. control + calcitriol; and ^§^ *P* < 0.05 vs. losartan. Note the increase in α-SMA and decrease in cubulin in losartan groups, which shows lack of cell differentiation.

**Figure 5:**
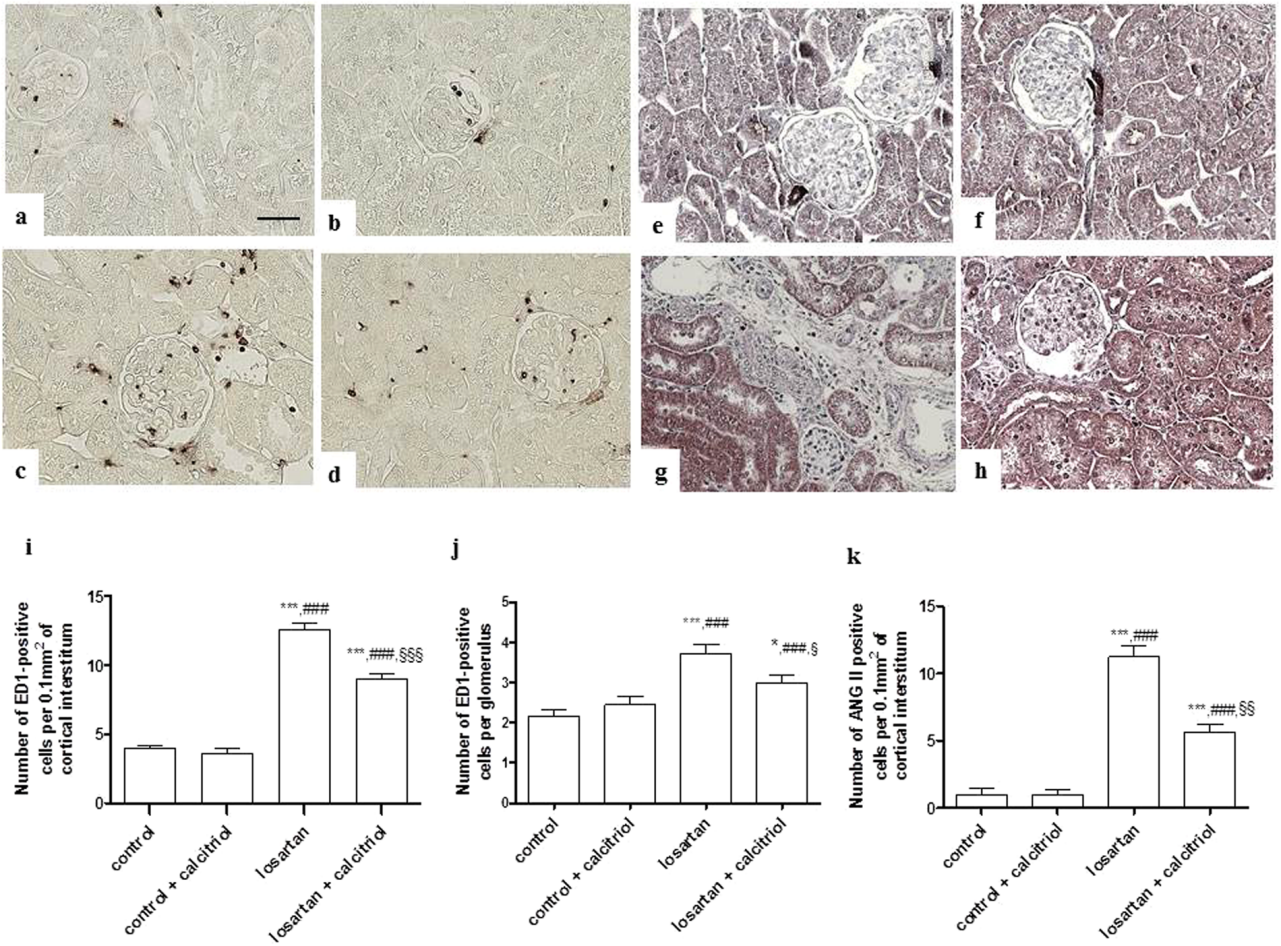
Immunolocalization of ED1-positive cells (macrophages/monocytes). (**a**–**d**) in the renal cortex and glomerulus and AngII (**e**–**h**) in the renal cortex of the control (**a**,**e**), control + calcitriol (**b**,**f**), losartan (**c**,**g**), and losartan + calcitriol (**d**,**h**) groups. The number of ED1-positive cells in the tubulointerstitial area from the renal cortex (**i**), glomerulus (**j**), and AngII in the renal cortex (**k**) of the control and experimental groups. Scale bar = 50 μm. Data are expressed as mean ± SEM (**i**). n = 7–12 animals per group. *P < 0.05 and ***P < 0.001 vs. control; ^###^ *P* < 0.001 vs. control + calcitriol; ^§^ *P* < 0.05, ^§§^ *P* < 0.01, and ^§§§^ *P* < 0.001 vs. losartan.

**Figure 6:**
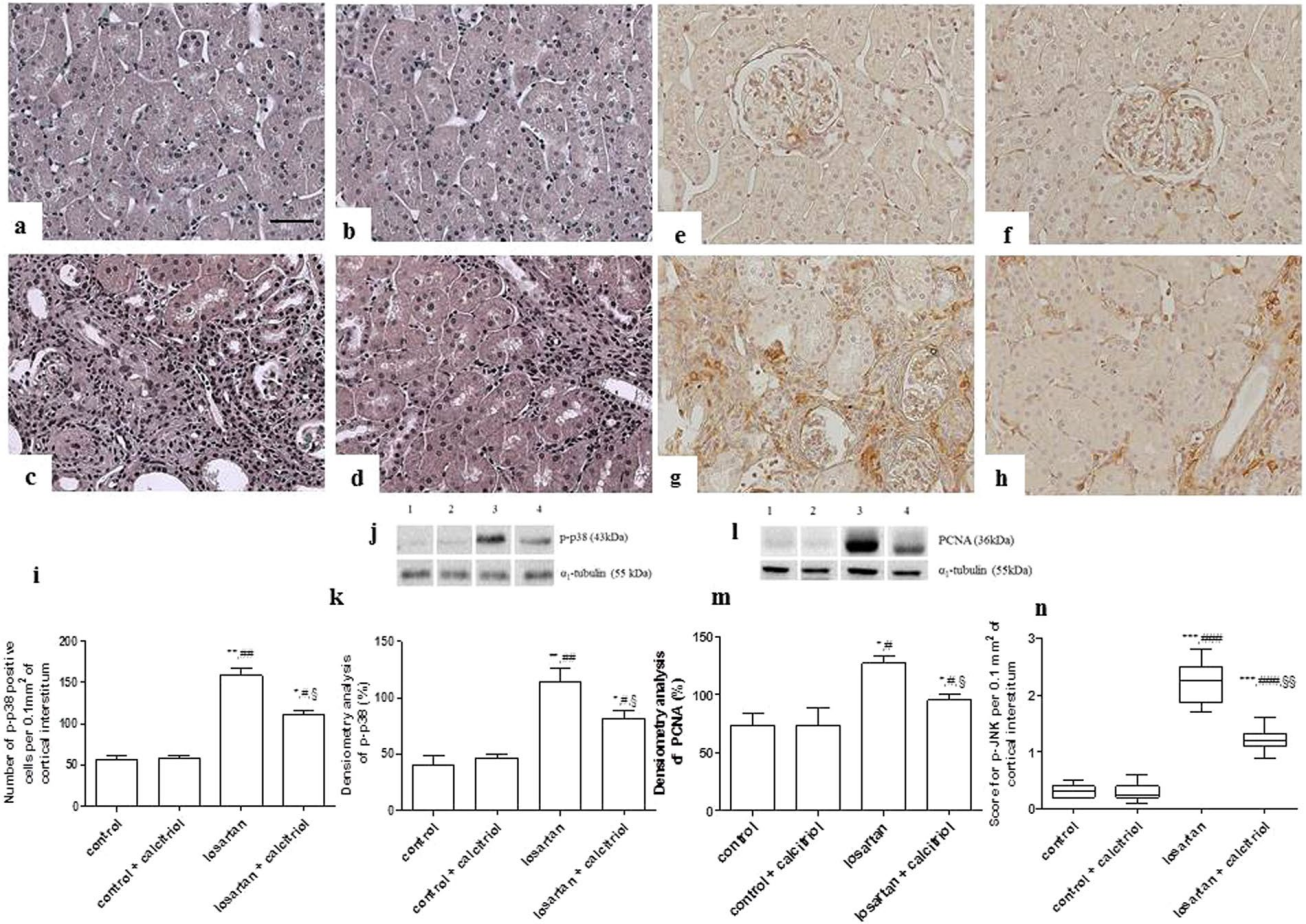
Renal cortical expression of p-p38 (**a**–**d**) and p-JNK (**e**–**h**) MAPKs in the renal cortex of the control (**a**,**e**), control + calcitriol (**b**,**f**), losartan (**c**,**g**), and losartan + calcitriol (**d**,**h**) groups. The number of p-p38-positive cells (**i**) and score for p-JNK (**n**) in the renal cortex. Scale bar = 50 μm. Western blot analysis of p-p38 and PCNA (**j**,**l**) in renal cortex from control (lane 1), control + calcitriol (lane 2), losartan (lane 3) and losartan + calcitriol (lane 4) groups. Densitometry of p-p38 and PCNA (**k**,**m**). A densitometric ratio between the densitometry of p-p38 or PCNA and α1-tubulin was calculated and data are expressed in comparison to the control, with the mean (±SEM) control value designated as 100%. Blots are representative images from independent experiments (n = 5–6 for each group). Data are expressed as median and interquartile range (25^th^–75^th^) (**n**) or mean ± SEM (**i**,**k**,**m**). n = 7–12 animals per group. *P < 0.05,**P < 0.01 and ***P < 0.001 vs. control; ^#^ *P* < 0.05, ^##^ *P* < 0.01 and ^###^ *P* < 0.001 vs. control + calcitriol; and ^§^ *P* < 0.05, and ^§§^ *P* < 0.01 vs. losartan.

Increased expression on vimentin was observed in the renal cortical tubular cells in the losartan-treated pups, showing lack of differentiation of these cells (Fig. 3a–d). This group also presented a higher expression of fibronectin in the tubulointerstitium from the renal cortex (Fig. 3e–h). Calcitriol treatment attenuated these alterations (Fig. 3i and l). The immunohistochemical analysis for α-SMA, another marker of lack of cell differentiation, showed an intense expression in the tubulointerstitial compartment and in the periglomerular parietal area in the animals exposed to losartan (Fig. 4a). In the control animals, α-SMA expression was confined to the arterial smooth muscle cells. The decrease in cubulin receptor expression also demonstrates the lack of differentiation of proximal tubule cells from the apical region of the kidney from these animals treated with losartan (Fig. 4c). Treatment with calcitriol decreased α-SMA expression and increased cubulin receptor expression (Fig. 4b,d), demonstrating its role in re-establishing cell differentiation (Fig. 4e,f). An inflammatory process evidenced by high infiltration of macrophages (ED1-positive cells) in both the tubulointerstitial and glomerular compartments of the kidneys from the losartan-treated rats was also observed (Fig. 5a–d). The number of AngII and p-p38-positive cells (Figs 5e–h and 6a–d), as well as the score for p-JNK (Fig. 6-n), were also increased in the cortical tubulointerstitial area of these animals. Calcitriol treatment prevented macrophage infiltration in both tubulointerstitial and glomerular compartments (Fig. 5i,j), and increased expression of AngII (Fig. 5k), p-p38, and p-JNK in the tubulointerstitium provoked by losartan (Fig. 6i–n). Higher expression of PCNA in the cortical tubulointerstitium was observed in losartan group (11.95 ± 0.64 per 0.100 mm^2^) compared to controls (3.62 ± 0.26) and calcitriol-treated animals (3.23 ± 0.24). This increase was attenuated in the losartan + calcitriol group (8.67 ± 0.47) (*P* < 0.001).

### Western Blot

Western blot analysis demonstrated the presence of 54 kDa (vimentin), 42 kDa (α-SMA), 43 kDa (p-p38), and 36 kDa (PCNA) protein lanes in the tissue samples from the renal cortex of all groups studied. The increase in the expressions of these proteins induced by losartan was attenuated by treatment with calcitriol. There was no difference in the intensity of the lanes for α1-tubulin between the different groups, showing the equivalence of protein loading and transfer (Figs 3j,k, 4g,h, 6j,k and 6l,m).

### TGF-β and MCP-1 urinary excretion

Increased urinary TGF-β and MCP-1 excretion were observed in the losartan group when compared to the control groups, which were attenuated by calcitriol treatment (Table 2).

**Table 2:** Urinary excretion of monocyte chemoattractant protein-1 (MCP-1) and Transforming growth factor beta (TGB-ß).

Groups	TGF-β (pg mg urinary creatinine^−1^)	MCP-1 (pg mg urinary creatinine^−1^)
Control	347 ± 67.5	527 ± 60.7
control + calcitriol	312 ± 62.6	445 ± 61.5
Losartan	2660 ± 490.7***^,###^	3430 ± 311***^,###^
losartan + calcitriol	1727 ± 204.9*^,#,§^	2100 ± 228*^,#,§^

Data are expressed as mean ± SEM. n = 7–12 animals per group. *P < 0.05 and ***P < 0.001 vs. control; ^#^ *P* < 0.05 and ^###^ *P* < 0.001 vs control + calcitriol; and ^§^ *P* < 0.05 vs. losartan.

## Discussion

Our data show that pups from mothers that received losartan during lactation presented a decrease in the glomerular area, tubular lumen dilation, and papillary atrophy, as well as an increase in the relative interstitial area of the renal cortex with inflammatory infiltration and fibrosis. These pups also showed higher liquid intake, lower body weight, and higher mortality rates. These findings were also observed in previous studies^21, 22^. The animals exposed to losartan presented several alterations in renal function, with decreased creatinine clearance, and urinary osmolality and increased fraction of sodium and potassium excretion. The higher liquid intake in the losartan group could be a consequence of the higher urinary volume of these rats. These alterations were associated with renal vessel malformation in the inner medulla and tubulointerstitial compartments of the renal cortex, medullary atrophy and a reduction of the glomerular area. Treatment with calcitriol attenuated several changes caused by exposure to losartan, except for urinary volume, osmolarity, and medullary atrophy. It was shown that AngII increases the capacity of the ureteric bud branches *in vivo* and is responsible for the formation of renal vessels during kidney development^23^. The integrity of these vascular structures and of the renal medullary parenchyma is fundamental for urinary concentration.

Morphometric studies showed a higher frequency of smaller (immature) glomeruli in the kidneys of rats from the losartan group compared to the control and losartan + calcitriol groups. The reduction of the glomerular area in the animals exposed to angiotensin (AT1) receptor antagonists was also observed by others authors^24, 25^. Treatment with calcitriol reduced the percentage of both smaller and larger glomeruli in rats exposed to losartan. The role of calcitriol in restoring cell differentiation was also evaluated by immunohistochemical and Western blot studies. The results showed that the increase of α-SMA labeling in the tubulointerstitial area and in the periglomerular parietal cells, and of vimentin in the tubule cells of the kidneys from losartan-treated rats, was attenuated by calcitriol treatment. Rats exposed to losartan also showed reduction of cubulin in the cells of proximal tubules, the receptor responsible for resorption of Vitamin D, compared to the controls. These alterations can be related to a lack of differentiation of these tubular cells^9–13^. Treatment with calcitriol attenuated α-SMA expression and increased cubulin receptor expression, demonstrating its role in cell differentiation. In recent years, clinical and experimental studies have shown the participation of calcitriol in cell differentiation and in the restoration of cell differentiation^26^. Calcitriol, when coupled with its Vitamin D receptor (VDR) forming the calcitriol/VDR complex, can cause specific actions even before cell differentiation begins. It enhances the expression and/or activity of various brush border enzymes, induces the formation of microvilli, and plays a role in cellular hierarchical organization^27, 28^.

An increase in the inflammatory process and in the expression of AngII in the renal cortex was observed in the animals exposed to losartan, which was attenuated by calcitriol treatment. Increased infiltration and activation of macrophages is one of the pathways by which AngII contributes to renal function disorders in renal pathologies^29^. Blockade of the AT_1_ receptor in the presence of higher levels of AngII has been shown to increase AT_2_ receptor stimulation^30^, which might at least partially explain our results. It has also been observed that the inflammatory and fibrogenic effects of AngII can be mediated by AT_2_ receptors, and that AT_2_ receptor expression was increased in renal tissues in this model^5–8^. The reduction of these inflammatory cells and the relative cortical interstitial area of calcitriol + losartan group was associated with decreased expression of mitogen-activated protein kinases (MAPK) (p-JNK and p-p38) and PCNA, as well as urinary levels of TGF-β and MCP-1, which are important markers of inflammation and cell proliferation. Data from experimental and clinical studies^31–33^ showed that calcitriol and its analogs can protect the kidneys from the renal damage observed in the progression of kidney disease through its effects in the local RAS and the MAPK pathway involved in these pathologic processes. Calcitriol is involved in the regulation of the renin gene expression^20^. Tian *et al*.^34^, in a study of diabetic nephropathy, observed the correlation between calcitriol in the downregulation of some inflammatory cytokines such as MCP-1 and TGF-β. The therapeutic effects of calcitriol are also related to its ability to preserve tubular epithelial integrity by epithelial-mesenchymal transition (EMT) inhibition^31^.

It was shown that in chronic kidney disease patients the decrease in GFR is associated with increased parathyroid hormone (PTH) levels^35^. Increased levels of PTH may play an important role in the fibrotic and inflammatory process. High levels of PTH were associated with raised MAPK phosphorylation, leading to increased proliferation, with more ECM components synthesis, with a consequent tubulointerstitial fibrosis^36^. The losartan group had decreased GFR, and thus possibly increased PTH levels. Some of the improved kidney parameters in the losartan + calcitriol group could be accounted for calcitriol effect in decreasing PTH levels.

Animals exposed to losartan during postnatal development present an increase in SBP in adult life^24–37^, as observed in the present study. The incidence of hypertension is higher among the human population that was born with renal malformation^38^. The increase in SBP observed after lactation in our model can also be explained by the increase in AngII due to the compensatory increase in renin synthesis^39^. Losartan blocking AT1 during renal development will cause an interruption in this negative feedback loop, leading to the overexpression of the renin gene and an increase in AngII. Calcitriol acts as a negative regulator of renin gene expression via inhibition of the cAMP response element-binding protein (CREB), which is required for the expression of renin^18–20^. The animals of the losartan + calcitriol group presented an attenuation of SBP increase that may be related to the action of calcitriol in the modulation of RAS and of the glomeruli and renal tubular cells differentiation of these animals. The rats exposed to losartan during lactation also presented albuminuria, although of low intensity. Treatment with calcitriol in our study reduced albuminuria in the animals exposed to losartan. A large number of studies have confirmed the potent antiproteinuric effect of calcitriol in several models of renal disease^40, 41^. The relationship between calcitriol deficiency and increased albuminuria was demonstrated in the third National Health and Nutrition Examination Survey (NHANES III), suggesting an antiproteinuric role of calcitriol^42^. The lack of differentiation of the proximal tubule cells with the decrease of cubilin expression, together with proteinuria, could impair the process of reabsorption and activation of Vitamin D.

In conclusion, our results demonstrate that treatment with calcitriol attenuated the alterations in renal function and structure provoked by losartan during lactation. These effects were associated with an increase in cell differentiation from the kidneys of these animals, and with a decrease in cell proliferation and inflammation.

## Materials and Methods

### Animals and experimental protocols

Experiments were performed on Wistar pups (male) from 12 pregnant females (Animal House of the Campus of Ribeirão Preto, University of São Paulo, Ribeirão Preto, SP, Brazil). The animals had free access to water and standard rat chow and were housed at standard room temperature (22 °C) chamber with a 12-h light/dark cycle. For mating, each male was housed with 3 females, and the first gestation day was determined based on the presence of copulatory plugs^5, 9^. Within 24 h of birth, the litters were reduced to 6 male pups to ensure equal feeding and divided into the following groups: (1) offspring of mothers treated with 2% sucrose solution (losartan diluent) (n = 24) and (2) offspring of mothers treated with losartan (All Chemistry, Brazil; 100 mg kg^−1^ day^−1^, diluted in 2% sucrose) (n = 30). Losartan or the sucrose solution was administered daily as a substitution drinking water during lactation (21 days).

After lactation, the rats were divided into 4 groups: (1) control (n = 12), (2) control + calcitriol (n = 12) (pups treated [or not] with calcitriol from dams that received 2% sucrose solution), (3) losartan (n = 14), and (4) losartan + calcitriol (n = 16) (pups treated [or not] with calcitriol from dams that received losartan). Calcitriol (6 ng/day, Calcijex, Abbott Laboratories, Chicago, IL, USA) was introduced on day 30 after birth and continued until day 60, administered by a mini-osmotic pump (Model 2004, Alzet, Cupertino, CA, USA). This dose of calcitriol was selected according to Kuhlmann *et al*.^43^. These authors demonstrated the beneficial effect of calcitriol in a subtotal nephrectomy model in rats.

Food consumption and liquid intake were measured in dams during the time of lactation (days 7 to 15) and in pups during days 40 to 46 according to previous study from our laboratory^9^. The liquid intake was measured every 24 h, measuring the difference between the amount of water offered and the amount remaining the next day, using a graduated test tube. The food consumption intake was measured by the difference in feed weight offered and consumed in 24 h.

The experiments were performed in accordance with the ethical principles for animal experimentation adopted by the Brazilian College of Animal Experimentation, and the Animal Experimentation Committee of the University of São Paulo at Ribeirão Preto Medical School approved the study protocol (COBEA/CETEA/FMRP-USP, Protocol no. 178/2014).

### Renal function studies

At 59 days old, the pups were placed in metabolic cages for 24-h urine sample collection to measure albumin by electroimmunoassay using a specific antibody against rat albumin; the data were expressed as urinary albumin 24 h^−1^ ^44^. The determination of urine osmolality was performed using an osmometer (Fiske OS Osmometer, Advanced Instruments, Norwood, MA, USA).

Sixty days old rats were anesthetized (sodium thiopental, 40 mg kg^−1^, i.p.), the aorta artery was cannulated, and blood samples were collected. Creatinine was measured using a commercial kit (Labtest Diagnostica S.A., Lagoa Santa, Brazil) in order to evaluate the Glomerular Filtration Rate (GFR) and sodium and potassium was measured using a 9180 Series electrolyte analyzer (Roche, Vienna, Austria) for plasma and urine samples.

The kidneys were removed and fixed in methacarn solution for 24 h, rinsed in ethanol (70%), and embedded in blocks of paraffin for histological and immunohistochemical studies. Urine was collected from the bladder were immediately treated with 1 mM phenylmethylsulfonyl fluoride (Sigma-Aldrich, St. Louis, MO, USA) and stored at −70 °C until used.

### Determination of systolic blood pressure

SBP was analyzed in the 60-day-old pups while conscious using the tail-cuff method (CODA System, Kent Scientific, Torrington, CT, USA). The animals were submitted to preconditioning 4–5 days before the procedure^45^.

### Quantification of TGF-β and MCP-1 in urine

TGF-β and MCP-1 quantification was performed using ELISA (Enzyme-Linked Immunosorbent Assay) commercial kits (Promega Corporation, Madison, WI, USA, and Pierce Biotechnology, Rockford, IL, USA, respectively). The median values of TGF-β and MCP-1 in the urine samples were expressed in picogram pg of TGF-β or MCP mg creatinine^−1^.

### Renal tissue morphometric analysis

The glomerular area of the 60-day-old animals was determined in 50 grid fields of the renal cortex (measuring 0.100 mm^2^), and the mean counts per 0.100 mm^2^ of the renal cortex were calculated for each rat. To analyze the planar glomerular area and the interstitial relative area of the renal cortex, the outer edges of all glomerular tufts and the interstitial area of each kidney were traced on a video screen, and the encircled areas were established by computerized morphometry (AxioVision Rel. 4.3; Zeiss, Jena, Germany). The relative interstitial area of the renal cortex was determined by dividing the interstitial area of 30 grid fields measuring 0.100 mm^2^ by the area of the total cortex determined in these grid fields.

### Immunohistochemical studies

Renal tissue was deparaffinized using xilol and submitted to immunohistochemical studies. Nonspecific antigen binding was blocked by incubation for 20 min with normal goat serum. The sections were incubated with anti-ED1 (1:1,000; Serotec, Oxford, UK), anti-cubilin (1:1500; Santa Biotechnology, Santa Cruz, CA, USA), or anti-vimentin antibodies (1:500; Dako Corporation, Glostrup, Denmark) for 60 min at room temperature; or with α-SMA (anti-α SMA, 1:1,000; Dako Corporation, Glostrup, Denmark), anti-phospho-p38 (p-p38; 1:1,000; Sigma-Aldrich, St. Louis, MO, USA), anti-proliferative cell nuclear antigen (PCNA, 1/1000; Sigma-Aldrich, St. Louis, MO, USA), anti-phospho-JNK (p-JNK; 1:30; Santa Cruz Biotechnology, Santa Cruz, CA, USA), anti-fibronectin (Chemicon International Inc., Temecula, CA, USA), or anti-AngII (1/200; Peninsula Laboratories, San Carlos, CA, USA) antibodies overnight at 4 °C^5, 9^. The avidin-biotin-peroxidase complex (Vector Laboratories, Burlingame, CA, USA) was used to detect the reaction products. The sections were then counterstained with methyl green or hematoxylin, dehydrated, and mounted.

In order to analyze α-SMA, vimentin, cubilin, p-JNK, and fibronectin staining, each glomerulus or cortical field (measuring 0.100 mm^2^ each) was semiquantitatively graded and the mean score per kidney was calculated^46^. The scores mainly reflected changes in the extent rather than in the intensity of staining, and depended on the percentage of the glomerulus or grid field showing positive staining: 0 = absent or <5% staining; 1, 5–25%; 2, 25–50%; 3, 50–75%; and 4, >75% staining^46, 47^. The numbers of ED1-, PCNA-, p-p38-, or AngII-positive cells in each glomerulus or cortical interstitial 30 grid field were determined in the renal cortex, and the mean counts were calculated for each kidney^5, 9^.

### Western blot studies

The tissue from renal cortex was homogenized in lysis buffer (50 mM Tris–HCl, pH 7.4, 150 mM NaCl, 1% Triton X-100, 0.1% SDS, 1 μg/mL aprotinin, 1 μg/mL leupeptin, 1 mM phenylmethylsulphonyl fluoride, 1 mM sodium orthovanadate, pH 10, 1 mM sodium pyrophosphate, 25 mM sodium fluoride, 0.001 M EDTA, pH 8) at 4 °C^48, 49^. Proteins (50 µg) were separated by sodium dodecyl sulfate polyacrylamide gel electrophoresis, transferred to nitrocellulose membranes, incubated for 1 h in blocking buffer (PBS, 5% skim milk), washed in buffer (TBS, 0.1% Tween 20, pH 7.6) and incubated with anti-vimentin (1:1,000; Dako Corporation, Glostrup, Denmark), anti-α-SMA (anti-α SMA, 1:1,000; Dako Corporation, Glostrup, Denmark), anti-phospho-p38 (p-p38; 1:1,000; Sigma-Aldrich, St. Louis, MO, USA), or anti-proliferative cell nuclear antigen (PCNA, 1/500; Sigma-Aldrich, St. Louis, MO, USA) antibodies, overnight at 4 °C^5, 9^. To adjust the equivalence of protein loading and/or transfer, the membranes were also incubated with anti-α_1_-tubulin monoclonal antibody (1/4,000; Sigma Chemical Co, St. Louis, USA) overnight at 4 °C. Blots were washed and incubated with horseradish peroxidase-conjugated goat anti-rabbit IgG (1/5000; Dako, Glostrup, Denmark) or anti-mouse IgG (1/10000; Dako, Glostrup, Denmark) for 1 h at room temperature. Membranes were then washed and membrane-bound antibodies were detected using the Supersignal West Pico Chemiluminescent Substrate (Pierce Chemical, Rockford, USA). The intensity of the identified lanes was quantified by densitometry using ImageJ NIH image software (http://www.nih.gov) and was reported in arbitrary units^9^. Protein estimations were performed using the Bradford method^50^.

### Statistical analysis

A nonparametric Kruskal-Wallis test followed by Dunn post-test was used to analyze the data that were not normally distributed, that were expressed as medians and interquartile ranges (25–75%). The analysis of variance with the Newman-Keuls multiple comparisons test was used for the analysis of normally distributed data. Statistical analyses were performed using the STATISTICA program version 10 (StatSoft, Tulsa, USA). These data were expressed as the mean ± standard error of the mean (SEM). In all cases, the level of significance was set at *P* < 0.05.
